# Spatial effects of air pollution on the economic burden of disease: implications of health and environment crisis in a post-COVID-19 world

**DOI:** 10.1186/s12939-022-01774-6

**Published:** 2022-11-15

**Authors:** Xiyu Zhang, Qi Xia, Yongqiang Lai, Bing Wu, Wanxin Tian, Wenqing Miao, Xinglin Feng, Ling Xin, Jingying Miao, Nianshi Wang, Qunhong Wu, Mingli Jiao, Linghan Shan, Jianzhao Du, Ye Li, Baoguo Shi

**Affiliations:** 1 Research Center of Health Policy and Hospital Management, School of Health Management, Harbin Medical University, Harbin, 150086 Heilongjiang China; 2 School of Public Health, Peking University, Beijing, China; 3 Department of Social Medicine, School of Health Management, Harbin Medical University, Harbin, Heilongjiang China; 4 The Department of Hospital Offices, Wuxi No.2 People’s Hospital, the affiliated Wuxi No.2 People’s hospital of Nanjing Medical University, Liangxi District, Wuxi, China; 5 Department of Economics, School of Economics, Minzu University of China, Beijing, 100081 China

**Keywords:** Air pollution, Economic burden of disease, Catastrophic health expenditure, COVID-19, Health service, Policy

## Abstract

**Background:**

Air pollution has been identified as related to the diseases of susceptible population, but the spatial heterogeneity of its economic burden and its determinants are rarely investigated. The issue is of great policy significance, especially after the epidemic of COVID-19, when human are facing the joint crisis of health and environment, and some areas is prone to falling into poverty.

**Methods:**

The geographical detector was adopted to study the spatial distribution characteristics of the incidence of catastrophic health expenditure (ICHE) for older adults in 100 rural areas in China at the prefecture-city level. The health factors, sociological factors, policy factors and environmental factors and their interactions are identified.

**Results:**

First, most health service factors had strong explanatory power for ICHE whether it interacts with air pollution. Second, 50 single-factor high-risk areas of ICHE were found in the study, but at the same time, there were 21 areas dominated by multiple factors.

**Conclusion:**

The different contributions and synergy among the factors constitute the complex mechanism of factors and catastrophic health expenditure. Moreover, during this process, air pollution aggravates the contribution of health service factors toward ICHE. In addition, the leading factors of ICHE are different among regions. At the end, this paper also puts forward some policy suggestions from the perspective of health and environment crisis in the post-COVID-19 world: environmental protection policies should be combined with the prevention of infectious diseases; advanced health investment is the most cost-effective policy for the inverse health sequences of air pollution and infectious diseases such as coronavirus disease 2019 (COVID-19); integrating environmental protection policy into healthy development policy, different regions take targeted measures to cope with the intertwined crisis.

**Supplementary Information:**

The online version contains supplementary material available at 10.1186/s12939-022-01774-6.

## Introduction

Countries around the world have been facing the threat of the coronavirus disease 2019 (COVID-19) pandemic. Most countries took measures to block or restrict travel and worked to curb the spread of the disease. Although this is a great crisis in human history, it has unintentionally helped people in some respects. The concentration of some air pollutants, such as NO_2_, has plummeted in China due to the COVID-19 lockdown, which has further positively affected people’s public health [1]. In the context of the 2020 COVID-19 pandemic, a study with interesting results estimated that 19,600 (15,300 to 24,000) PM_2.5_-related excess mortality was avoided in China during the blockade period up to 15 May 2020 [2].

Several studies have confirmed the relationship between air pollution and respiratory [3–7], cardiovascular, cerebrovascular [8–10], and other diseases [11–13] in the past. Recently, many articles are focusing on air pollution and individual economic burden [14, 15]. However, when the economic burden of disease exceeds the residents’ affordability, this kind of medical expenditure becomes catastrophic and may directly lead to disease-related poverty. Obviously, the economic burden of disease is an absolute value, arrived at without considering the income difference of different groups, so it is impossible to directly measure the risk of health poverty, that is, high economic burden of disease does not necessarily cause serious economic consequences. In addition, the family provides financial support for each member, and the economic support among family members implies that when describing the economic risk of disease, the individual economic burden of disease is far less accurate than the catastrophic health expenditure (CHE) at the family level.

While tackling air-pollution-related diseases, more attention should be paid to individuals vulnerable to CHE. First, compared with urban residents, rural residents are more likely to suffer from diseases caused by air pollution due to more outdoor work, less awareness of self-protection measures, and limited access to medical services. Comparing the cause-specific mortality between urban and rural areas in China from 2014 to 2016, the relative risks of all-cause, cardiovascular, and respiratory mortality in rural areas were 1.01, 1.03, and 1.04 times higher than those in urban areas, respectively [16]. Moreover, rural residents have more out-of-pocket expenses than urban residents. Taking China’s data in 2014 as an example, the rural out-of-pocket ratio was 63.4%, while it was 55.5% for urban residents [17]. A Turkish study showed that the risk of CHE in rural families is 2.5 times higher than that in urban families [18]. Second, whether facing air pollution or not, the elderly are vulnerable toward CHE. Several studies found that CHE was most common in families with people aged 60 or over [19–21]. Some people believe that getting older in age will increase medical expenditure [22, 23], while others believe that age has no direct relationship with medical expenditure, but the elderly are often closer to death and tend to incur high medical expenditure [24, 25]. Moreover, the risk of heart disease due to air pollution increases sharply with age [26]. At the same time, the sick elderly cannot work, causing a certain economic burden to the family and society at large [27]. Older adults are also proven to be more vulnerable to air pollution, and more studies regarding the formulation of efficient medical policy is required [28].

In addition, despite the spatial spill over effect of air pollution, few relevant studies use spatial methods to analyse the correlation between various related factors [29–31]. To the best of our knowledge, only a few studies [32] have focused on the impact of air pollution on government health expenditure at the provincial and country levels. We use CHE as a measure of the economic risk of disease, which occurs when the total amount of family out-of-pocket expenses equals or exceeds 40% of the family’s ability to pay for costs other than living expenses [33]. A new spatial analysis method, the geographical detector, was adopted to study the spatial stratified heterogeneity and driving factors of air pollution affecting the incidence of catastrophic health expenditure (ICHE) for rural older adults to provide a guiding conclusion for the public health protection policy after the lifting of the lockdown.

## Theoretical framework

Health service factors are closely related to CHE. According to their characteristics, we divided the various health service factors into three categories: demand, supply, and utilisation of health services. The demand for health services is mainly reflected by health status, which the prevalence of non-communicable diseases (NCDs) and prevalence of disability (DA) in this study belong to. Li et al. [34] found that the probability of CHE of residents suffering from NCDs in rural areas has greatly increased, and Ethel Mary Brinda [35] confirmed that the economic burden of the disabled is greater. In fact, the expanding demand for health services requires more utilisation of health services, which eventually aggravates the economic burden of diseases. The supply of health services reflects its availability, where number of hospital beds per ten thousand people (NHB), number of hospitals (NH), and number of doctors per thousand people (ND) are commonly used as measurement indicators. Access to health services is associated with ICHE [36, 37]. Some countries, such as the United Republic of Tanzania and Zambia, are unable to receive medical treatment nearby due to geographical healthcare accessibility and need to spend extra money in certain situations, such as transportation, which increases indirect medical expenses [36] and further increases the economic burden. The utilisation of health service included utilisation rate of outpatient service (UROS), utilisation rate of hospitalisation (URH), out-of-pocket payment (OOP), inpatient and outpatient medical expenses (IOEXP). It is the actual utilisation of health services, which is closely related to the economic burden of diseases. Health expenditure generated by health services can be divided into three categories based on the source of financing: society, government, and individual. The former two can be expressed as insurance, while the latter, that is, out-of-pocket expenses, become the actual economic burden of disease faced by individuals. CHE is also judged by comparing the relationship between out-of-pocket expenses and the capacity to pay [32]. Some studies have found that due to the more expensive medical expenses and insufficient reimbursement level, a high utilisation rate of health services [38] aggravates the disease economic burden of individuals [37].

**Fig. 1:**
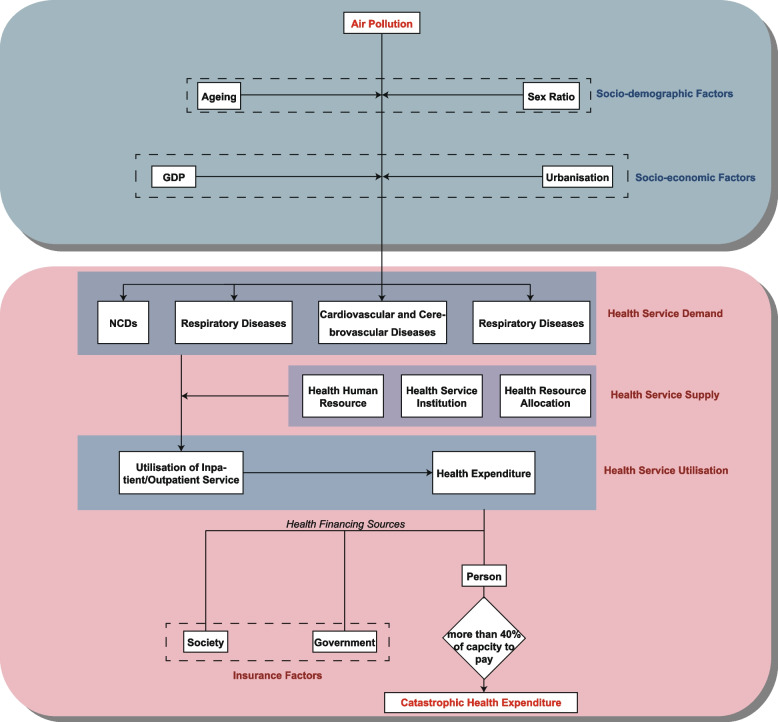
Influence mechanism of each factor on ICHE

The synergy among other factors constitutes the complex mechanism of air pollution and catastrophic health expenditure, as shown in Fig. 1. In the process, vulnerable populations such as older adults [39], women [40], and the disabled [41] are more sensitive to diseases caused by air pollution. Disease reflects the demand for health services, which needs to be accompanied by the actual supply of health services [42] to lead to the actual utilisation of health services. In addition, the role of the economy cannot be underestimated. It not only affects the potency of air pollution, but also changes the medical habits of residents, thereby increasing the utilisation of health services. Economic factors influence this mechanism through multiple channels and stages. Economic development has improved residents’ living standards, improved their health status, increased health investment (investment in health care), and even indirectly affected health through education. At the same time, economic development has also brought about environmental pollution and ecological damage (air pollution is one of them) and increased the demand for health services (the consequences of lifestyle changes such as obesity, smoking, alcoholism and lack of exercise, modern social diseases, mental health problems, negative social events, socio-demographic characteristics, and changes in the disease spectrum). Poor health may also curb economic development. The most common reasons are the reduction of high-quality labour force, disease loss, and economic cost. In conclusion, it is not difficult to imagine that air pollution is not only one of the consequences of economic development, but also a major factor increasing the damage of economic development to residents’ health levels and the economic burden of disease.

## Materials and methods

### Material

#### Air quality index

The Ministry of Environmental Protection (MEP) of the People’s Republic of China (PRC) has employed the new index “air quality index (AQI)” since January 2013. Our study used this composite index to describe regional air quality. AQI considers six major air pollutants in China, namely, PM_2.5_, PM_10_, SO_2_, NO_2_, O_3_, and CO, and comprehensively evaluates China’s air quality.

The data source of AQI in this study is China National Environmental Monitoring Centre (CNEMC). CNEMC covers four levels: national, provincial, municipal and county, including 1436 monitoring stations in 338 cities above prefecture level, 96 regional stations, and 15 background stations [43]. The original AQI data is derived daily from various monitoring points in the country. After data processing, the annual average data of each region is formed.

#### CHARLS data

China Health and Retirement Longitudinal Study (CHARLS) is a longitudinal survey designed to represent mainland China residents aged 45 and over. There is no upper age limit. It attempts to establish a high-quality public micro database, which can provide a wide range of information from socio-economic status to health status to meet the needs of scientific research of the elderly. CHARLS coordinates with leading international research in the Health and Retirement Research model to ensure the adoption of best practices and international comparability of results. The national baseline survey was conducted from 2011 to 2012: the second wave in 2013, the third wave in 2015, and the fourth wave in 2018. To ensure the representativeness of the sample, the CHARLS baseline survey covered 150 countries/regions and 450 villages/urban communities and involved 17,708 individuals from 10,257 families, reflecting the overall situation of China’s middle-aged and elderly population. At present, the first four waves of the CHARLS data and life history have been publicly released. In this study, the latest 2018 CHARLS database was used as the primary data source, but finally, due to the small sample size of individual research areas, only 100 sample cities (a list of these 100 regions is provided in Appendix Table 4) after data processing, including municipalities, were retained for the sake of the reliability of the research results.

#### Dependent and independent variables

Based on the original CHARLS data, we used the method provided by the World Health Organisation to calculate the incidence of CHE. The standard of CHE is out-of-pocket expenses of ≥40% of the family’s capacity to pay [32]. The independent variables in this study include AQI, sex ratio (SR), ageing (AG), gross domestic product (GDP), urbanisation rate (UR), insurance coverage rate (ICR), OOP, IOEXP, UROS, URH, NHB, ND, NH, NCDs, and DA. Although some of the independent variables are from CHARLS, these indicators are mainly from the yearbooks of cities in China, as shown in Table 1 (descriptive statistics are presented in Appendix Table 5).

**Table 1:** Research Indicators and data sources

Variable	Symbol	Description	Source
Explained variable	ICHE	Incidence of catastrophic health expenditure	CHARLS
Core explanatory variable	AQI	Air Quality Index	China National Environmental Monitoring Centre, CNEMC
Socio-demographic factor	SR	Sex Ratio	CHARLS
	AG	Ageing, proportion of families with older adaults (> = 65)	CHARLS
Economic factor	GDP	Gross domestic product (hundred million CNY)	China City Statistical Yearbook-2019
	UR	Urbanisation rate	China City Statistical Yearbook-2019
Insurance factor	ICR	Insurance coverage Rate	CHARLS
Health service factor	OOP	Out-of-pocket payment (CNY)	CHARLS
	IOEXP	Inpatient and outpatient medical expenses (CNY)	CHARLS
	UROS	Utilisation rate of outpatient service	CHARLS
	URH	Utilisation rate of hospitalisation	CHARLS
	NHB	Number of hospital beds per ten thousand people	China City Statistical Yearbook-2019
	ND	Number of doctors per thousand people	China City Statistical Yearbook-2019
	NH	Number of hospitals	China City Statistical Yearbook-2019
	NCDs	Prevalence of non-communicable diseases	CHARLS
	DA	Prevalence of disability	CHARLS

### Data processing

In this study, the study area was bounded by the administrative boundary, so we first used ArcGIS10.2 to transform the study area to 20 km × 20 km grid points for further data input. Geographical detectors are good at analysing type variables, so we then discretise the 15 other types of variables in this study and perform statistical analysis. The method adopted here is the natural breakpoint method provided by ArcGIS 10.2 software.

### Geographical detector

Considering the dimension of space, the assumption of Independent and Identically Distributed required by classical statistics is often not tenable. Spatial autocorrelation, spatial heterogeneity, and modified areal unit problem need to be considered. To solve these issues separately, different spatial methods based on different assumptions are proposed. China’s economic development stratification may further lead to regional differences in medical insurance, and therefore, our study mainly focuses on spatial stratified heterogeneity. A geographical detector [44, 45] is a new statistical method to measure spatial differentiation and attribute its driving factors; this method does not require a linear assumption and has clear physical meaning. The basic idea is to divide the research area into several strata. If the sum of the variance of the strata is less than the total variance of the region, it is considered that there is spatial stratified heterogeneity; if the spatial distribution of the two variables is coupled, measured by the geographical detector q statistic, there is a statistical association between the two variables. In the cross-sectional study, geographical detectors can suggest potential causality more strongly, because the probability of consistent distribution of two variables in two-dimensional space is far lower than that in one-dimensional curves. When the linear regression is significant, the geographical detector must be significant, and when the linear regression is not significant, the geographical detector may still be significant. Geographical detectors also have lower requirements for large samples and can still obtain reliable estimation results even when the number of observations is below 30. In addition, a geographical detector is immune to the collinearity between variables and able to effectively detect the real interaction between two variables not limited to the multiplicative interaction [46, 47]. Geographical detectors include factor, interactive, risk, and ecological detectors. In this study, we used factor, interactive, and risk detectors. In addition, ecological detectors mainly focus on whether there are significant differences between the two factors on the spatial distribution of ICHE, which is not beneficial to this study and thus not applicable.

#### Factor detector

The spatial differentiation of ICHE is measured and attributed by the q statistic:

$$\textrm{q}=1-\frac{\sum_{\textrm{h}=1}^{\textrm{L}}{N}_h{\sigma}_h^2}{N{\sigma}^2}$$

where *h* = 1, …, *L* refers to the strata of ICHE or its factor X; *N*_*h*_ and *N* are the number of units of stratum *h* and the whole region, respectively; *and σ*_*h*_^*2*^ and *σ*^*2*^ are the variances of the ICHE value of stratum *h* and the whole region, respectively.

The q value was between 0 and 1. The closer the q value is to 1, the more obvious the spatial stratified heterogeneity of the ICHE. If the stratification is partitioned by the independent variable X, the closer the q value is to 1 and the stronger the explanatory power of the independent variable X to attribute the ICHE. The explanation strength was 100 × q%.

#### Interactive detector

Identify interactions between different risk factors.

The process of the interactive detector is to calculate the q value of the two factors to ICHE, then calculate the q value of the two factors when they interact, and finally compare the two q values. The relationship between the two factors can be divided into five types, as shown in Table 2.

**Table 2:** Types of interaction between two independent variables and dependent variables

Criterion	Interaction
q(x_1_,x_2_) < Min(q(x_1_),q(x_2_))	Nonlinear weakening
Min(q(x_1_),q(x_2_)) < q(x_1_,x_2_) < Max(q(x_1_),q(x_2_))	Single-factor nonlinear weakening
q(x_1_,y) > Max(q(x_1_),q(x_2_))	Bi-enhancement
q(x_1_,y) = q(x_1_) + q(x_2_)	Independent
q(x_1_,y) > q(x_1_) + q(x_2_)	Nonlinear enhancement

#### Risk detector

To focus on whether there is a significant difference in the mean value of ICHE between the two sub-regions.

The significance of the data was analysed according to the classification method mentioned above:

4 $${t_{\overline{y}}}_{h=1\ {\overline{y}}_{h=2}}=\frac{{\overline{Y}}_{h=1}-{\overline{Y}}_{h=2}}{{\left[\frac{Var\left({\overline{Y}}_{h=1}\right)}{\eta_{h=1}}+\frac{Var\left({\overline{Y}}_{h=2}\right)}{\eta_{h=2}}\right]}^{{}^{1}\!\left/ \!{}_{2}\right.}}$$

where ${\overline{Y}}_h$ is the mathematical expectation of Y in the sub-region (h), η_h_ is the number of samples in the sub-region (h), and Var is the variance.

## Results

### Spatial distribution characteristics of ICHE

**Fig. 2:**
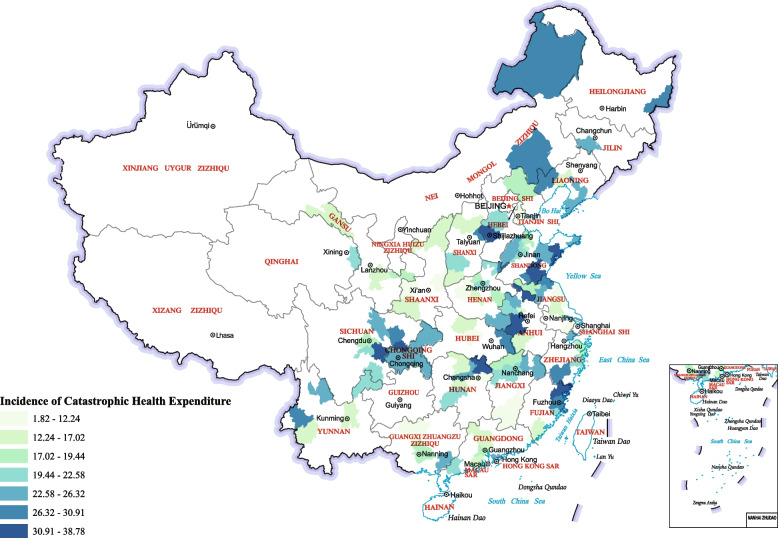
Spatial distribution of ICHE

China is composed of 333 prefectures. The distribution of cities with high ICHE is dotted in the rural areas of China investigated in this study. As shown in Fig. 2, 40 cities with high ICHE (> 22.58) are scattered across China’s four major economic regions [48]: east (15), west (12), middle (9), and northeast (5). Although the agglomeration phenomenon is not obvious in the economic zone, it is not the same among provinces. Of the cities with high ICHE, 17.5% are located in Shandong Province (Eastern Economic Zone), 15% in Sichuan Province (Western Economic Zone), and 7.5% in Anhui Province (Central Economic Zone).

In addition, Lu’an City’s ICHE (38.78%) was the highest, and Chaozhou City’s ICHE (1.82%) was the lowest, with a difference of 21.3 times.

### Results of geographical detector

The geographical detector analysed the previous raster data and obtained the following results:

#### Factor detector

As shown in Table 3, all 15 dependent variables had significant explanatory power for ICHE. Among the 15 dependent variables involved in this study, OOP (30.82%), IOEXP (26.4%), AG (17.88%), GDP (17.44%), DA (15.45%), NCDs (14.7%), and AQI (14.68%) had stronger explanatory power for ICHE. In addition, from the perspective of dimensions, except for AG (social demographic factor), GDP (economic factor), and AQI (air pollution), these factors are all health service factors, which include the demand, supply, and utilisation of health services.

**Table 3:** Results of factor detector

	OOP	IOEXP	AG	GDP	DA	NCDs	AQI	URH	SR	NH	ND	UR	NHB	UROS	ICR
**q statistic**	0.3082	0.2640	0.1788	0.1744	0.1545	0.1470	0.1468	0.1344	0.1300	0.1254	0.1099	0.1042	0.0862	0.0815	0.0709

The q statistics of all dependent variables were significant at 99% level (*P* < 0.01)

#### Interactive detector

**Fig. 3:**
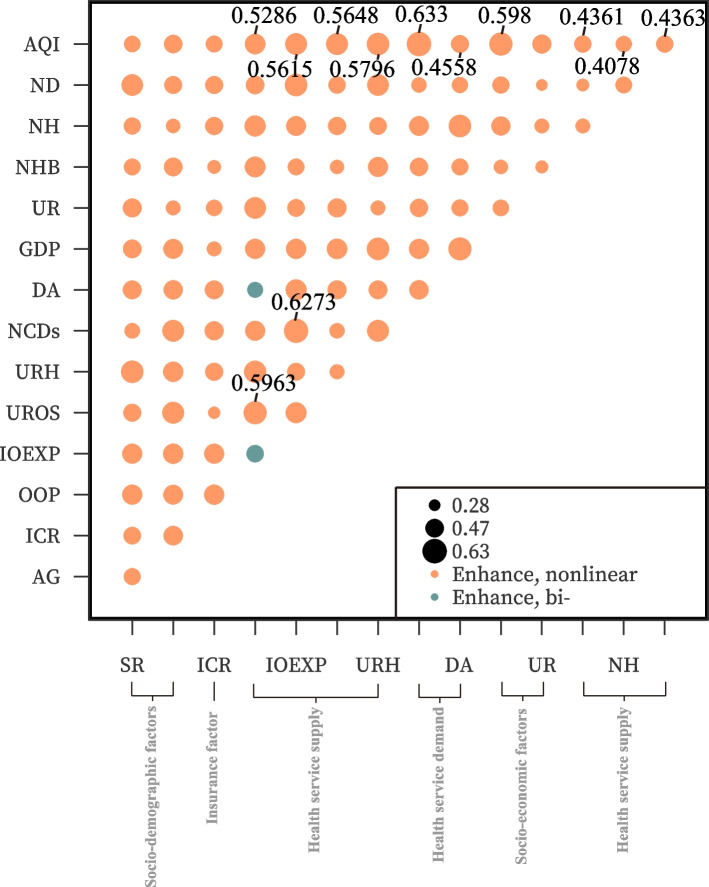
Results of interactive detector

The results in Fig. 3 show that the explanatory power of many groups after the interaction of two factors exceeds 50%, such as IOEXP and NCDs (62.73%), and UROS and OOP (59.63%). However, in this study, our main concern was the interaction between air pollution and other factors. It should be noted that the interaction between AQI and the other 14 independent variables is a nonlinear enhancement, that is, factors have increased the explanatory power for the occurrence of ICHE caused by AQI, and the q value after interaction is greater than the sum of q values under the independent conditions. When AQI interacted with some of these factors, the explanatory effect exceeded 50%, such as NCDs (63.3%), GDP (59.8%), URH (57.96%), UROS (56.48%), IOEXP (56.15%), and OOP (52.86%). We found that the factors with strong explanatory power when interacting with air pollution are all health service factors, except for GDP, which is an economic factor.

#### Risk detector

**Fig. 4:**
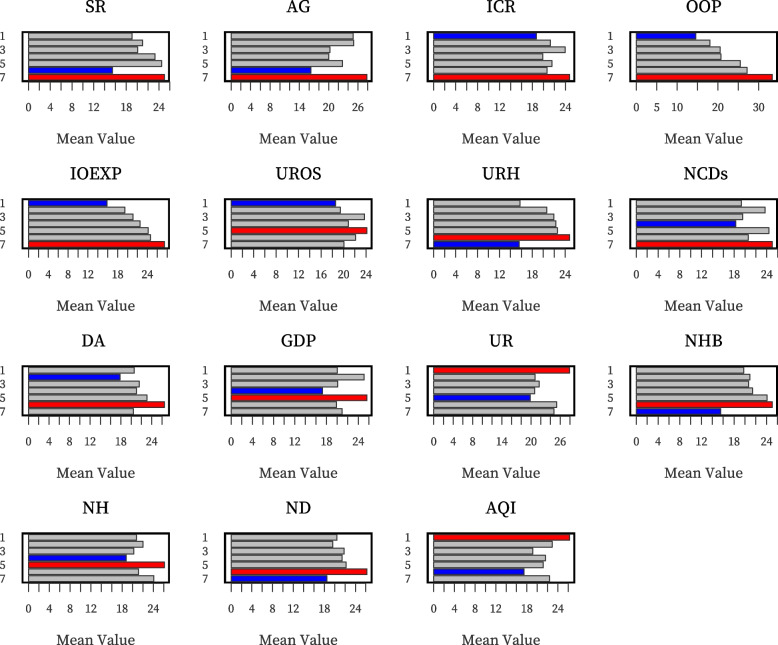
Result of risk detector

**Fig. 5:**
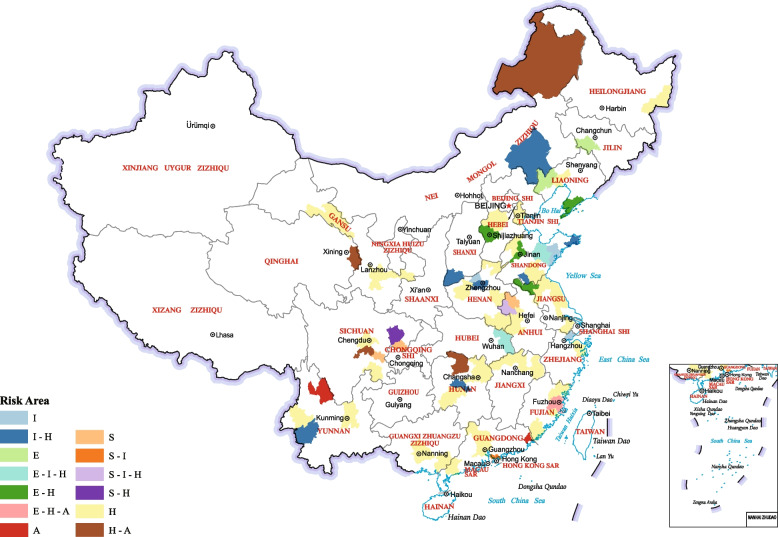
Spatial distribution of high-risk areas

A risk detector is used to explore which category of a risk factor corresponds to the high or low value of the dependent variable ICHE. By calculating the mean value of ICHE in each stratum, we observed that ICHE changed with the increasing risk factors. In Fig. 4, the numbers 1–7 represent the seven classifications of the natural breakpoint method from small to large (strata 1 represents the lowest quantity level). Figure 4 shows the ICHE mean values corresponding to different factors at different quantity levels. ICHE reached the maximum value in the 7th stratum of IOEXP, AG, NCDs, SR, OOP and ICR, but reached the maximum value in the 1st stratum of AQI and UR. However, this does not mean that the relationship between the variables must be linear and that higher ICHE appears in the 7th strata of UR. Moreover, ICHE has reached the maximum value in the relatively high quantity levels of GDP, DA, URH, NH, ND, NHB, and UROS. The areas with high ICHE may be caused by a single- or multi-dimensional risk factor. To simplify the expression, we symbolised air pollution (A), socio-demographic factors (S), economic factors (E), insurance factors (I), and health services factors (H). Through risk detection, we found that there are 50 single-factor risk areas in 100 sample cities, including H risk areas (40), I risk areas (3), S risk areas (3), A risk areas (2), and E risk areas (2). For example, air pollution is the only leading factor in Chaozhou, while health services are the only leading factor in Guangzhou. Seventeen double-factor risk areas were also found, including the I-H risk area (7), E-H risk area (4), H-A risk area (4), S-I risk area (1), and S-H risk area (1). Changde and Hulunbuir are two H-A risk areas. In addition, there are four treble factor risk areas, including the E-I-H risk area (2), S-I-H risk area (1), and E-H-A risk area (1). The situation in these areas is dominated by three factors, such that Fuzhou, an E-H-A risk area, is more complicated than the former two (Fig. 5).

## Discussion

### Air pollution factor aggravates the contribution of health service factors to ICHE

Although the factor detector found that all factors had a significant impact on the spatial distribution of ICHE (*P* < 0.01), the explanatory power of OOP, IOEXP, UROS, URH, NCDs, DA, NHB, NH, and ND for the spatial distribution of ICHE reached 30.82%, 26.4%, 8.15%, 13.44%, 14.7%, 15.45%, 8.62%, 12.54%, and 10.99%, respectively. Most health service factors have strong explanatory power for ICHE. Furthermore, when interacting with air pollution, these explanatory powers increased to 52.86%, 56.15%, 56.48%, 57.96%, 63.3%, 45.58%, 43.61%, 40.78%, and 43.63%, respectively. It shows that the health service factor contributes more to ICHE in the context of air pollution and always plays an especially important role. Furthermore, we have tentatively introduced annual average PM_2.5_ data from CNEMC to test the robustness of AQI related results. The results showed that there was little difference between the results of PM_2.5_ and that of AQI (Appendix Fig. 1), indicating that AQI related results were robust.

As shown in Fig. 1, the emergence of air pollution further damages public health. Through multi-directional infiltration and transmission, the high incidence of air pollution-related diseases increases the demand for health services. This increase in demand may pose a significant challenge to the limited health service resources in a region. Once the government fails to bear the cost of the increase of relevant personnel and high-tech in the supply of health services, a large part of it will be transferred to the patients, which constitutes the main economic burden of the disease. In addition, it makes the existing facilities and medical technicians unable to meet the needs of residents, affecting the geographic accessibility to health care [49], forcing the patient to go farther to access health services, and resulting in significant indirect medical expenses. Moreover, no matter what the reason is, the increase in health service supply will eventually be partly passed on to the residents, making them bear more medical costs. Even if the health service resources in this area are sufficient, the increased demand for health services will be transformed into a scenario of more utilisation of health services and generation of more economic burden of disease. The quantitative relationship between health service factors and air pollution levels has been verified in many studies. A study of Xi’an urban residents found that when the concentration of PM_2.5_ increased by 100 μg/m^3^, the mortality of chronic obstructive pulmonary disease increased by 7.25% [50]. Another study in Hong Kong showed that the relative risk (RR) of hospital admissions for chronic obstructive pulmonary disease was 1.031 per 10 μg/m^3^ increase in PM_2.5_, at lag days ranging from lag zero to cumulative lag 0–5 [51].

### The leading factors of ICHE are different among regions, which is the scientific basis for policymaking

Among the 71 high-risk areas mentioned above, there were 50 single-factor high-risk areas, but at the same time, there were 21 areas dominated by multiple factors. These multi-factor high-risk areas include I-H risk area (7), E-H risk area (4), H-A risk area (4), S-I risk area (1), S-H risk area (1), E-I-H risk area (2), S-I-H risk area (1) and E-H-A risk area (1). We will focus on Chaozhou (A risk area) and Fuzhou (E-H-A risk area). Chaozhou is a coastal city in the eastern Guangdong Province. Its terrain is high in the northwest and low in the southeast. It is surrounded by mountains on three sides and the sea on one side. It is quite easy to form a stable atmospheric environment in Chaozhou, but also prone to serious air pollution problems [52], which also confirms why Chaozhou is listed as a high-risk area of ICHE dominated by air pollution. Fuzhou, as the starting point of the maritime Silk Road, has more complex leading factors in the ICHE. First, the distribution of health services in Fuzhou is uneven, mainly concentrated in the Gulou and Taijiang Districts, and its overall development cannot keep up with the changes in population [53]. We assume that, in the context of air pollution, this fierce conflict will be further intensified, and residents will have to pay extra for medical treatment. This also explains why health services have become one of the leading factors of ICHE in the region. Second, the seasonal characteristics of air pollution in Fuzhou were obvious. Tropical cyclones and mild subtropical high make air pollutants diffuse or settle in warm period, and the ‘temperature inversion’ phenomenon during cold periods and fireworks during Spring Festival produce added air pollutants, which are difficult to diffuse [54]. Although air pollution is not particularly serious, its special seasonal characteristics may make its impact on the ICHE more obvious in the region. Finally, thanks to the support of the government, Fuzhou has undergone long-term friendly economic exchanges with Taiwan since 2006. The Minjiang River has become a new zone of development. However, economic development has damaged local land cover [55], which can no longer purify air. In addition, with economic development, the adjustment of industrial structures and process of industrialization has been deteriorating the air quality of Fuzhou [56]. As a result, Fuzhou’s air pollution may cause serious health consequences and increase the economic burden of diseases. The leading factors of these high-risk areas reflect that policymaking should follow the characteristics of the region, and targeted policies can reduce the economic burden of the residents using half the effort.

### Early warning on disease economic burden: formulating health and environmental policies after COVID-19

In the previous section, we described the impact of air pollution on the spatial distribution of ICHE, and extensive studies have focused on the health consequences of air pollution and proposed environmental and health policies for air pollution. However, it is far from sufficient. Human activities have brought about an increasing number of climate problems and public health emergencies. In the face of continuous environmental deterioration and catastrophic public emergencies such as COVID-19, the spill over and dissemination of the two create a linked destiny—having to face the common problems of economic stagnation and damage to human health.

As we all know, both COVID-19 and air pollution are caused by human activities that lead to environmental degradation, resulting in avoidable loss of life and economic losses, mainly due to delays, inadequacies, and mistakes in action [57]. Health problems and air pollution are intertwined, which fully exposes the sluggish and inadequate response of existing policies [58]. We expect the two problems to be studied as a whole and not in isolation in future research. Our study has tentatively proposed a direction for solving the above problems, especially in the aftermath of the COVID-19 pandemic, where various factors have deteriorated to varying degrees. We are trying to predict the severity of this problem considering the COVID-19 epidemic by a tentative exercise using educated guesses and aim to further solve these environmental health related problems from a broader and more comprehensive perspective, which may reflect the policy value of this study.

#### Environmental protection policies should be combined with the prevention of infectious diseases

The pandemic prompted countries to suspend economic activities and establish temporary isolation policies, further leading to the drop in air pollution before and after the pandemic. NO_2_ dropped by 12·9 μg/m^3^ and PM_2.5_ dropped by 18·9 μg/m^3^ in most cities in China due to the quarantine period during the COVID-19 pandemic. The short-term improvement of air quality during the quarantine period avoided a total of 8911 NO_2_-related deaths and 3214 PM_2.5_-related deaths [59] and limited the pandemic to some extent [60]. However, these improvements are not reliable in the long term, as they are based on the sacrifice of economic activities. Once the quarantine is lifted, the air pollution situation may further deteriorate, which may lead to more serious diseases and corresponding economic risks according to our results of risk detector.

In past management, most people did not realise a connection between air pollution and virus transmission. Relevant research on the outbreak of COVID-19 has paid attention to this matter. It has been found that a large number of bacteria and viruses are suspended in PM_2.5_, PM_10_, and other atmospheric particles in air pollution; that is, viruses such as the SARS-CoV-2 can be transmitted to the human body through biological aerosols [61]. First, this discovery should help policymakers realise that it is extremely important to reduce pollution sources as much as possible, because a clean natural environment can also reduce the burden of infectious [62] and other [11, 12] diseases caused by air pollution. Second, an appropriate increase in environmental protection policy funds can be used to avoid significant economic losses caused by air pollution and its related diseases. Finally, environmental governance goals may incorporate the prevention of infectious diseases in normalisation [63], erupting into a greater force. In this era of catastrophic infectious disease epidemics, paying attention to the positive impact of environmental protection in preventing infections is of paramount importance.

#### Advanced health investment is the most cost-effective policy for the adverse health consequences of air pollution and infectious diseases such as COVID-19

Both the pandemic itself and the butterfly effect brought about by the pandemic may cause significant changes across some factors in our model. Lockdowns during the COVID-19 pandemic may make it difficult to get nutritious food and physical activity [64], further increasing the prevalence of NCDs. According to the results of the risk detector, it may cause higher ICHE in China. Further, cancelling non-urgent outpatient visits and being infected with COVID-19 will worsen the condition of NCD patients, which causes more health expenditure. On the one hand, the progression of NCDs may have a negative effect on the outcome of the SARS-CoV-2 infection [65], but on the other, it has squeezed out the original primary health services and intensified the conflict between supply and demand. Although the total amount of health resources, such as the number of beds and doctors, has not decreased, the cost of healthcare for patients has further increased due to diversion or prohibition in the pandemic.

For a long time, compared with other aspects, politicians have regarded health as a secondary aspect of the government’s action plan, thus redistributing the budget and paying more attention to those aspects that drive economic development [61]. An important way to improve health services is to improve health investment [66]. A recent study found that the higher the government health expenditure, the lower the mortality brought on by the COVID-19 pandemic [61]. Health investment strengthens the health care sector’s capacity to respond to epidemics and other diseases [61], including those caused by air pollution, which can benefit residents facing the economic burden of any disease. However, we must emphasise that proactive health investment should be encouraged. One of the most important ways is to improve the infrastructure construction of health services, whether via diagnostic ability, the application of artificial intelligence technology and communication technology, or the allocation of medical technicians and hospital beds. It is worth noting that the government should be the main body of the above investment and should aim to allow residents to enjoy the health accessibility and health equity brought by health investment, rather than letting them pay for health investment. For example, in China, the government has made significant investments for constructing primary health institutions, providing residents with cheap, accessible, and efficient basic health services [67]. Moreover, additional government investment in health should be coordinated with other policies to make residents enjoy less of an economic burden, such as the price control of medical and health services. China’s health care reform has provided an example for a vast number of developing countries, by reducing drug prices, establishing a national essential drug system, and promoting the reform of compensation mechanisms in public hospitals [68]. All of these will play a role when air pollution or the follow-up of an outbreak of an epidemic can harm public health and economic burden. Health is not only the cornerstone of society, but also the result of social progress; health investment is also an investment in socioeconomic development.

#### Integrating environmental protection policy into healthy development policy: different regions take targeted measures to cope with the intertwined crisis

Whether it is an industrial policy or the follow-up recovery strategy of COVID-19, ‘people-oriented’ is the theme we emphasise. However, when people’s health and economic burdens are linked to air pollution, we must pay attention to environmental protection in some policies. COVID-19 has seriously damaged the global economy [69]. How to stimulate the economy to return to the normal track is a new topic of concern for policymakers. However, for health promotion, we suggest that every health policy should pay attention to environmental protection and seek a balance between the two. Both the Paris Agreement [70] and China’s new pledges towards carbon neutrality by 2060 remind us that environmental protection should be rooted in multi-field policies; it is an important part of the government’s health collaborative governance. The government’ decisions may reflect climate change policies in the coming decades and lay a foundation for sustainable progress in economic development and healthcare [71]. In addition, all regions should precisely diagnose the core driving force leading to the economic burden of disease, develop response models for different combinations of risk factors, and take targeted measures to cope with the health problems caused by intertwined crisis [71]. As for the economic burden of disease caused by different factors, local governments should be responsible for coordinating various departments to jointly govern health problems.

### Supplementary Information

**Additional file 1: Appendix Fig. 1.** The robustness test results of AQI related results.

## Data Availability

The data used in this study are proprietary data owned by Peking University. The data are not publicly available but are available to researchers from corresponding author by application to the Peking University for CHARLS Program (http://charls.pku.edu.cn/en).

## Appendix

Table 4

**Table 4:** Name list of 100 research regions

Region Name	Afillited Province
Anqing	Anhui
Anyang	Henan
Anshan	Liaoning
Baoji	Shaanxi
Baoding	Hebei
Baoshan	Yunnan
Binzhou	Shandong
Haozhou	Anhui
Changed	Hunan
Chaoyang	Liaoning
Chaozhou	Guangdong
Chengdu	Sichuan
Chengde	Hebei
Chifeng	Inner Mongolia
Dalian	Liaoning
Dezhou	Shandong
Dingxi	Gansu
Foshan	Guangdong
Fuzhou	Fujian
Fuyang	Anhui
Ganzhou	Jiangxi
Guangan	Sichuan
Guangzhou	Guangdong
Haidong	Qinghai
Hanzhong	Shaanxi
Hechi	Guangxi
Hulun Buir	Inner Mongolia
Huainan	Anhui
Huanggangshi	Hubei
Jixi	Heilongjiang
Jinan	Shandong
Jiaxing	Zhejiang
Jiangmen	Guangdong
Jiaozuo	Henan
Jinzhou	Liaoning
Jingmen	Hubei
Jiujiang	Jiangxi
Kunming	Yunnan
Lijiang	Yunnan
Lishui	Zhejiang
lianyungang	Jiangsu
Liaocheng	Shandong
Lincang	Yunnan
Linfen	Shanxi
linyi	Shandong
Luan	Anhui
Loudi	Hunan
Luoyang	Henan
Maoming	Guangdong
Meishan	Sichuan
Mianyang	Sichuan
Nanchang	Jiangxi
Nanchong	Sichuan
Nanning	Guangxi
Neijiang	Sichuan
Ningbo	Zhejiang
Ningde	Fujian
Pingdeshan	Henan
Pingliang	Gansu
Putian	Fujian
Puyang	Henan
Qingdao	Shandong
Qingyuan	Guangdong
Shangrao	Jiangxi
Shaoyang	Hunan
Shenzhen	Guangdong
Shijiazhuang	Hebei
Siping	Jilin
Suzhou	Jiangsu
Suqian	Jiangsu
Suzhou	Anhui
Taizhou	Zhejiang
Taizhou	Jiangsu
Tianjin	municipality directly under the Central Government
Weihai	Shandong
Weifang	Shandong
Weinan	Shaanxi
Xiangyang	Hubei
Xinzhou	Shanxi
Xinyang	Henan
Xuzhou	Jiangsu
Yancheng	Jiangsu
Yangzhou	Jiangsu
Yangquan	Shanxi
Yiyang	Hunan
Yibin	Sichuan
Yichun	Jiangxi
Yulin	Shaanxi
Yulin	Guangxi
Yueyang	Hunan
Yuncheng	Shanxi
Zaozhuang	Shandong
Zhangye	Gansu
Zhangzhou	Fujian
Changsha	Hunan
Zhaotong	Yunnan
Zhengzhou	Henan
Chongqing	municipality directly under the Central Government
Zhoukou	Henan
Ziyang	Sichuan

Table 5

**Table 5:** Descriptive statistics

Variable	Obs	Mean	Std. Dev.	Min	Max
ICHE	100	21.348	7.295	1.818	38.776
SR	100	.926	.354	.333	3
AG	100	41.545	11.097	0	81.818
ICR	100	97.228	2.519	83.919	100
OOP	100	700.013	588.735	214.149	5336.342
IOEXP	100	235.271	213.034	0	1136.797
UROS	100	16.723	7.094	4.255	33.333
URH	100	16.254	6.972	0	31.579
NCDs	100	47.175	34.27	0	100
DA	100	13.112	7.304	0	31.959
GDP	100	4231.491	5227.298	350.77	24,640.801
UR	100	56.573	14.401	21.234	99.75
NHB	100	45.296	11.375	18.516	84.996
NH	100	139.93	130.441	30	892
ND	100	2.469	.666	1.327	4.307
AQI	100	63.879	18.646	22.87	104.531

## Abbreviations

COVID-19: Coronavirus disease 2019
CHE: Catastrophic health expenditure
ICHE: Incidence of catastrophic health expenditure
NCDs: Non-communicable diseases
DA: Prevalence of disability
NHB: Number of hospital beds per ten thousand people
NH: Number of hospitals
ND: Number of doctors per thousand people
UROS: Utilisation rate of outpatient service
URH: Utilisation rate of hospitalisation
OOP: Out-of-pocket payment
IOEXP: Inpatient and outpatient medical expenses
MEP: Ministry of Environmental Protection
PRC: The People’s Republic of China
AQI: Air quality index
CNEMC: China National Environmental Monitoring Centre
CHARLS: China Health and Retirement Longitudinal Study
SR: Sex ratio
AG: Ageing
GDP: Gross domestic product
UR: Urbanisation rate
ICR: Insurance coverage rate
