# Supplementary figures and images for: Spatial effects of air pollution on the economic burden of disease: implications of health and environment crisis in a post-COVID-19 world
Source: Int J Equity Health. 2022 Nov 15;21:161. doi: 10.1186/s12939-022-01774-6 (PMC9664438; doi:10.1186/s12939-022-01774-6)

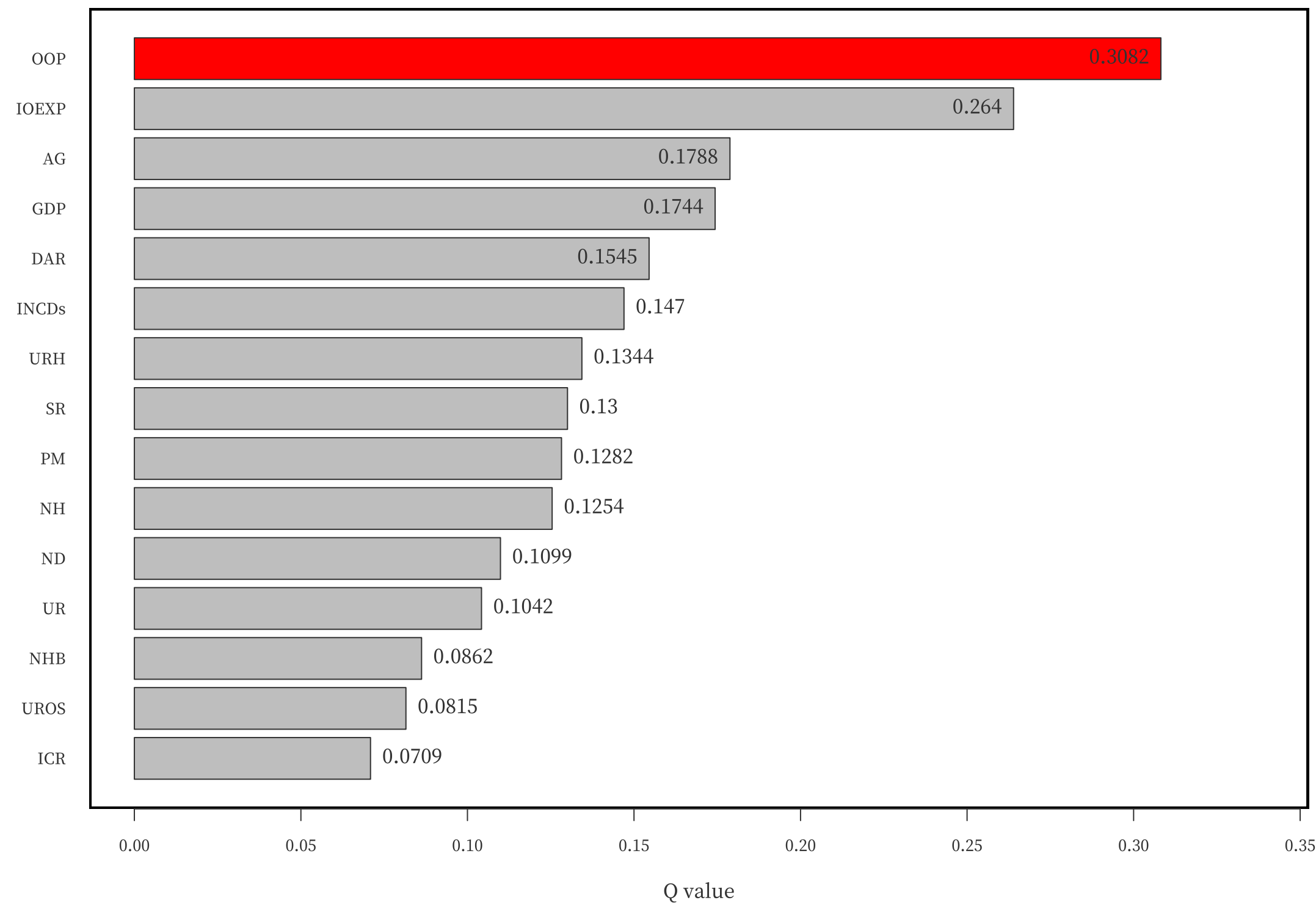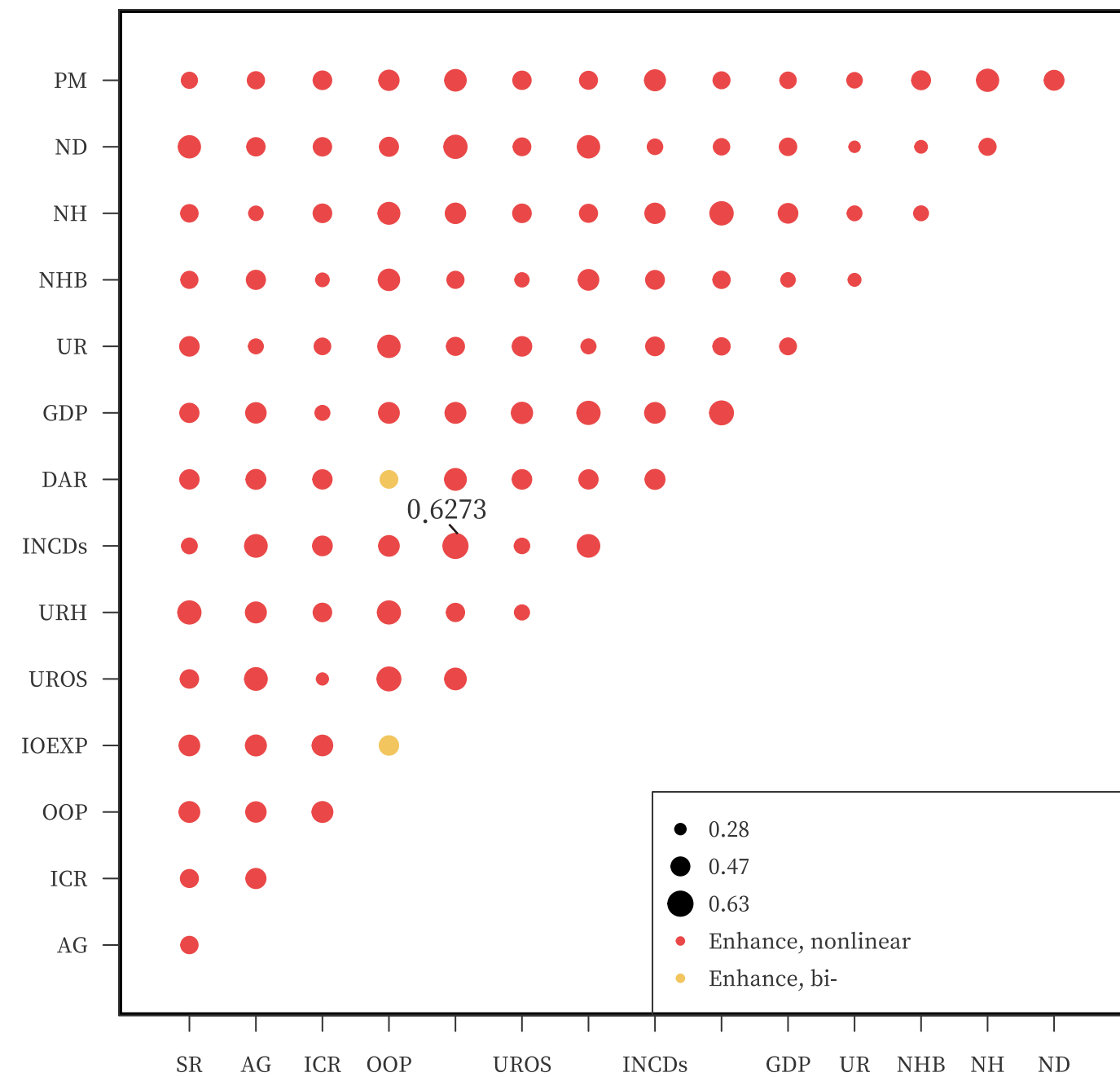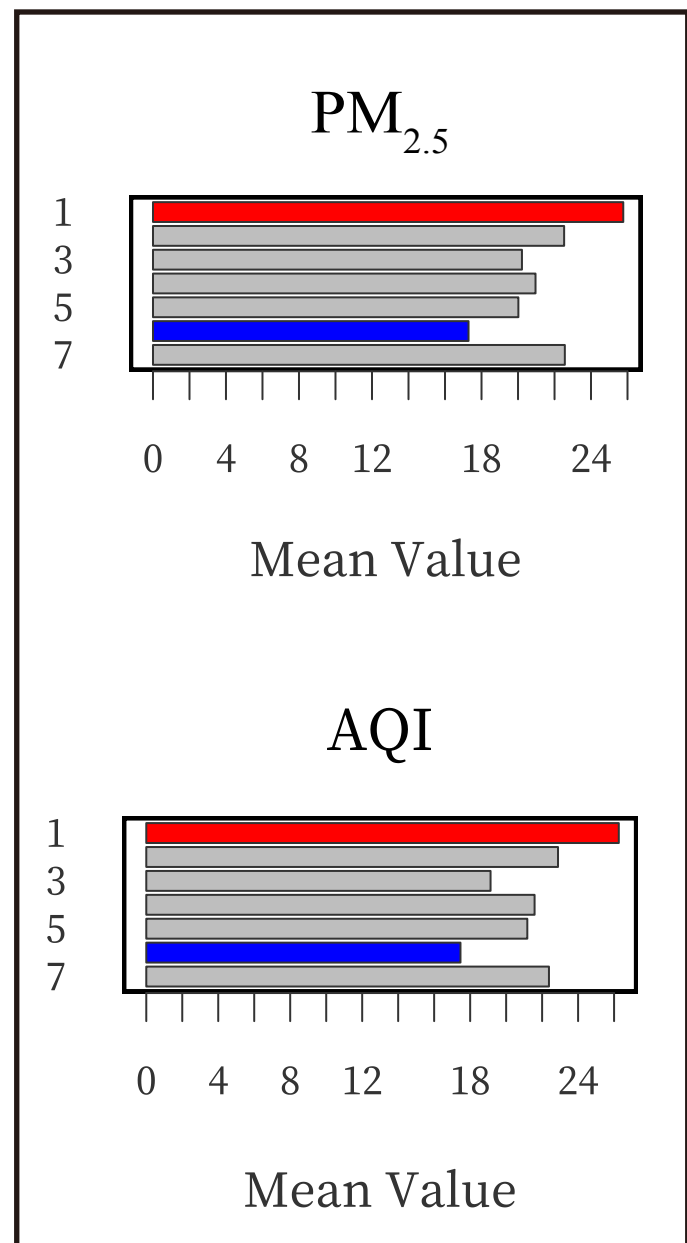

Supplement: Supplementary file 1 — Additional file 1: Appendix Fig. 1. The robustness test results of AQI related results. [file 12939_2022_1774_MOESM1_ESM.pdf]
